# Effects of Cinnamon Consumption on Glycemic Indicators, Advanced Glycation End Products, and Antioxidant Status in Type 2 Diabetic Patients

**DOI:** 10.3390/nu9090991

**Published:** 2017-09-08

**Authors:** Behrouz Talaei, Atieh Amouzegar, Shamim Sahranavard, Mehdi Hedayati, Parvin Mirmiran, Fereidoun Azizi

**Affiliations:** 1 Department of Clinical Nutrition and Dietetics, Faculty of Nutrition Sciences and Food Technology, National Nutrition and Food Technology Research Institute, Shahid Beheshti University of Medical Sciences, P.O. Box 19395-4741, Tehran, Iran; b.talaei@outlook.com; 2 Department of Nutrition, Faculty of Health, Kerman University of Medical Sciences, P.O. Box 19395-4741, Kerman, Iran; 3 Endocrine Research Center, Research Institute for Endocrine Sciences, Shahid Beheshti University of Medical Sciences, P.O. Box 19395-4763, Tehran, Iran; amouzegar@endocrine.ac.ir (A.A.); azizi@endocrine.ac.ir (F.A.); 4 Department of Traditional Pharmacy, School of Traditional Medicine, Shahid Beheshti University of Medical Sciences, P.O. Box 19395-4763, Tehran, Iran; ssahranavard@sbmu.ac.ir; 5 Cellular & Molecular Endocrine Research Center, Research Institute for Endocrine Sciences, Shahid Beheshti University of Medical Sciences, P.O. Box 1985717413, Tehran, Iran; hedayati47@yahoo.com

**Keywords:** type 2 diabetes, cinnamon, glycemic indices, inflammatory indicators

## Abstract

The aim of the current study was to determine the effect of a daily intake of three grams of cinnamon over eight weeks on glycemic indicators, advanced glycation end products, and antioxidant status in patients with type 2 diabetes. In a double-blind, randomized, placebo controlled clinical trial study, 44 patients with type 2 diabetes, aged 57 ± 8 years, were randomly assigned to take either a three g/day cinnamon supplement (*n* = 22) or a placebo (*n* = 22) for eight weeks. We measured the fasting blood glucose, insulin, hemoglobinbA1c, homeostasis model assessment for insulin resistance (HOMA-IR), carboxymethyl lysine, total antioxidant capacity, and malondialdehyde levels at the beginning and the end of the study. Thirty-nine patients (20 in the intervention group and 19 in the control group) completed the study. After an eight-week intervention, changes in the level of fasting blood glucose, insulin, hemoglobinbA1c, HOMA-IR, carboxymethyl lysine, total antioxidant capacity, and malondialdehyde were not significant in either group, nor were any significant differences between groups observed in these glycemic and inflammatory indicators at the end of the intervention. Our study revealed that cinnamon supplementation had no significant effects on glycemic and inflammatory indicators in patients with type 2 diabetes.

## 1. Introduction

Diabetes is one of the most common multifactorial public health diseases and a leading cause of death worldwide [1]. According to reports of the World Health Organization, the prevalence of diabetes in adults has been increasing due to dramatic changes in lifestyle, and it is projected to reach over 300 million cases by the year 2030 in both the developed and developing countries [2,3]. A previous survey in an Iranian population, aged 25–64 years, indicated that the national prevalence of diabetes is about 7–8% of the population [4]. Some metabolic disorders such as B-cell dysfunction, impaired insulin secretion, and insulin action contribute to the pathogenesis of diabetes. Several metabolic disturbances, including hyperglycemia, hyperinsulinemia, and inappropriate changes in metabolism of proteins, fats are the main characteristics of this disease [5,6]. 

Management of diabetes to prevent its complications and increase life expectancy and quality of life of diabetic patients becomes possible with appropriate intervention and modifications in lifestyle, including changes in dietary pattern, regular physical activity, and anti-diabetic medications [7,8]. Recently, studies investigated the potential protective effect of some herbal products on treatment of diabetes has been also assessed in human and animal models of type 2 diabetes [9].

Cinnamon (*Cinnamomum zeylanicum* Blume), as one of the common spices, contains various antioxidant compounds that are routinely used in a traditional system of medicine to treat chronic diseases such as cardiovascular diseases and diabetes in various regions of the world [10,11,12]. According to data available, the results of previous randomized controlled trials (RCTs) on the effects of cinnamon on glycemic parameters were inconsistent [12,13,14,15,16,17]. Some previous animal and human studies found that cinnamon intake can improve insulin resistance and decrease blood glucose concentrations and hemoglobinA1c (HbA1c) [12,14,18]. However, Vanschoonbeek et al. reported that cinnamon supplementation had no beneficial effects on fasting blood glucose (FPG) or insulin concentrations [17]. In addition, some others revealed no significant effect of cinnamon intake on HbA1c and fasting blood glucose [15,16]. However, it was likely that significant changes in these markers could be happened with longer follow-up.

Previous reporting indicated that oxidative stress and advanced glycation end products (AGEs) are key participants in the development and progression of type 2 diabetes and its complications [19]. On the contrary, it has been seen that the higher total plasma antioxidant capacity is significantly lower in diabetic patients rather than non-diabetic subjects [20]. To the best of our knowledge, to date, only a few RCTs have investigated the effects of cinnamon intake on advanced glycation end products, antioxidant capacity, and inflammatory markers, and they show conflicting results. In animal model studies, it has been seen that cinnamon improves antioxidant capacity with decreasing the malondialdehyde (MDA) levels [21,22] and decrease the formation of AGEs [23]. However, in Iranian women, taking cinnamon for six weeks did not have any significant effect on MDA levels [24].

Considering the limited studies available on the effects of cinnamon on managing and controlling type 2 diabetes, in the current study, we have investigated the effect of cinnamon supplementation on glycemic indices, AGEs, and antioxidant status in patients with type 2 diabetes. To do this, we performed a double-blind, randomized, placebo controlled clinical trial to assess the effect of three grams per day of cinnamon supplementation for eight weeks on FPG, insulin, HbA1c, HOMA-IR, carboxymethyl lysine, total antioxidant capacity, and MDA in 44 patients with type 2 diabetes.

## 2. Materials and Methods

### 2.1. Subjects

This is a double-blind, randomized, placebo controlled clinical trial conducted in Tehran province between 2016 and 2017 for which 44 patients, aged 25–70 years with type 2 diabetes, were randomly enrolled in two groups, the placebo and intervention. 

We used the formula for parallel clinical trials, considering type I error (α) of 0.05 and type II error (β) of 0.20 (power = 80%) to calculate the sample size of 20 for each group. However, assuming an estimated 10% dropout rate, there were 22 patients in each group (44 patients in total) to compensate for the probable loss to follow-up.

Inclusion criteria were: history of diabetes <8 years, body mass index (BMI) ranged 18.5–30 kg/m^2^, fasting blood glucose <180 mg/dL, 2 h blood glucose <250 mg/dL, glycemic control with metformin therapy, women without pregnancy and lactation, not having allergy to cinnamon, and free of some chronic diseases (including kidney, ischemic heart, or chronic inflammatory diseases, autoimmune disorders, chronic thyroid disease, stomach ulcers and infections, and insulin dependent type 2 diabetes). Exclusion criteria were noncooperation of patients during the study, pregnancy and lactation, detection of any side effects while taking cinnamon supplements, changes in the routine management of diabetes, drug abuse, alcohol and anti-inflammatory drug consumption.

### 2.2. Ethical Issues

The current study was conducted according to the principles of the Declaration of Helsinki, and the study protocol was approved by the Ethics Committee of the Shahid Beheshti University of Medical Sciences, Tehran, Iran (No.: IR.SBMU.NNFTRI.REC1394.36). We explained the study protocol and risks of experimental procedures carefully to all participants, after which their written informed consent was obtained. The study was registered at the Iranian registry of clinical trials, registration number IRCT2016061128392N1.

### 2.3. Study Design

**Figure 1:**
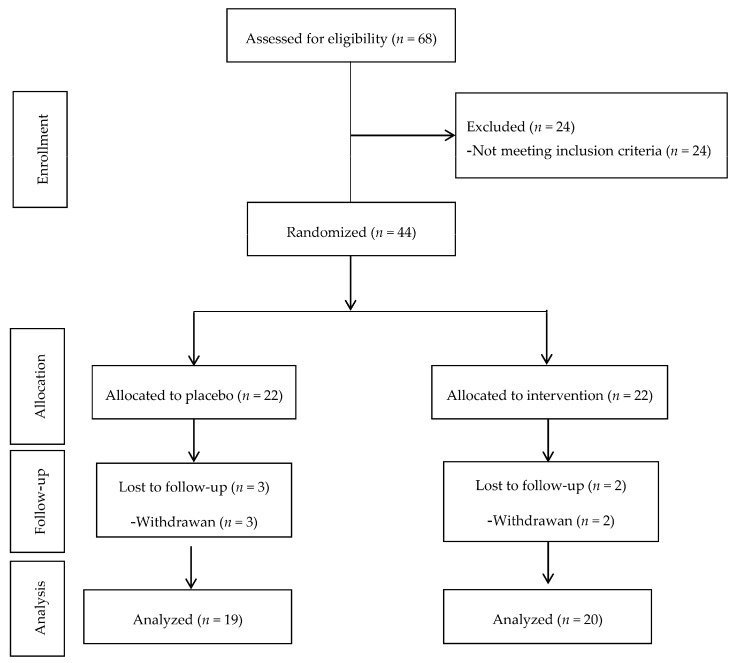
Summary of patient flow chart.

We selected eligible patients based on inclusion criteria from Endocrinology and Metabolism center, Shahid Beheshti University of Medical Sciences and randomly assigned them into two intervention groups, taking either cinnamon supplements (*n* = 22) or placebo (*n* = 22) for 8 weeks. Supplements and placebos were packaged in the same form and investigators and subjects were blinded to group assignment and to capsule content until the end of the analysis (Figure 1).

Preparation of supplements was performed with coordination and supervision of departments of traditional pharmacy, Shahid Beheshti University of Medical Science. First, the microbial quality of cinnamon and placebo was assessed based on world health organization (WHO) guidelines in the microbiology laboratory. Then, in the traditional medicine pharmacy, cinnamon sticks were finely ground. Cinnamon as treatment and also microcrystalline cellulose as placebo placed in capsules were not distinguishable from each other in color, smell, taste, shape, and size. Finally, the capsules were packaged and labeled equally. Each capsule contained either 1000 mg of cinnamon or microcrystalline cellulose. In the beginning and again at the fourth week of the study, the participants were given cinnamon supplements or placebos that were sufficient for use over four weeks and they were asked to consume one 1000 mg capsule of cinnamon or placebo after each main meal (breakfast, lunch and dinner) for eight weeks. In addition, patients were asked not to change their routine physical activity or normal dietary pattern and to continue their medications (metformin therapy) during the intervention.

We contacted the patients weekly to ensure that participants would act in compliance with the protocol of study, and remind them to take their supplements/placebos daily; they were also asked to return the original tablet bottles for capsule counts and assess compliance, so that the all unused capsules could be checked by counting the unconsumed capsules at each visit (every four weeks). 

### 2.4. Lifestyle and Anthropometric Assessment

The patients were interviewed by trained interviewers using pretested questionnaires for collection of socio-demographic and other information, including data on age, sex, education levels, marital status, employment status, duration of diseases, medication use and smoking at the beginning and end of the study. Weight was measured and recorded without shoes and wearing light clothing, using a digital scale with an accuracy of up to 100 g. Height was measured in a standing position without shoes, using a stadiometer with a minimum measurement of 5 mm. Body mass index (BMI) was calculated as weight in kilograms divided by height in meters squared.

### 2.5. Clinical and Biological Measurements

A 10 mL venous blood sample was taken after 12 h of overnight fasting in a sitting position according to the standard protocol and centrifuged within 30–45 min of collection at baseline and after the eight-week intervention. All blood analyses were performed on the day of blood collection at the laboratory of the Endocrinology and Metabolism center, Shahid Beheshti University of Medical Sciences.

Two mL of whole blood was added to a vial containing ethylenediaminetetraacetic acid (EDTA) anticoagulant for analyzing HbA1c level, immediately, and blood samples were centrifuged at room temperature for 10 min in a clinical centrifuge at 3000 rpm for plasma separation. Plasma samples were stored in a freezer at −70 °C for later analyses in micro tubes with a mL capacity. FPG was measured by the enzymatic colorimetric method with glucose oxidase, using commercial kits (Pars Azmoon, Tehran, Iran). The blood level of HbA1c was measured by high performance Ion Exchange Chromatography (IEC) method (ZellBio GmbH Kit, Ulm, Germany). Fasting plasma levels of insulin were analyzed using the auto-analyzer with 2 micU/mL sensitivity, by a commercially available enzyme-linked immunosorbent assay (ELISA) kit (Monobind, Commercial ELISA kit, Lake Forest, California, CA, USA). In addition, a commercial kit with 0.1 mmol sensitivity (ZellBio GmbH, Commercial ELISA kit, Ulm, Germany) was used for measuring of total antioxidant capacity. Plasma level of carboxymethyl lysine was measured ELISA method (ZellBio GmbH, Commercial ELISA kit, Ulm, Germany). We also determined the MDA concentration (μmol/L) by chromatography assay. Finally, the homeostasis model assessment for insulin resistance (HOMA-IR) was conducted according to their suggested formulas as follows [23,25]:

HOMA-IR = [fasting insulin (μU/mL) × fasting glucose (mmol/L)]/22.5

### 2.6. Statistical Analysis

Statistical analyses were conducted using the Statistical Package for Social Sciences (version 15.0; SPSS, Chicago, IL, USA) and *p*-values < 0.05 were considered statistically significant. To assess the normality of the variables, Kolmogorov–Smirnov analysis was used. The characteristics of the patients at baseline and at the end of the study are expressed as the mean ± SD for normally distributed contentious variables, median (25–75 inter-quartile range) for skewed continuous variables, and percentages for categorical variables. To compare quantitative variables between intervention and placebo groups, we used Student’s *t*-test and Mann–Whitney test for means and medians, respectively. In addition, a Pearson Chi-Square test was applied for comparison of qualitative variables between the two groups. Paired *t*-test was used to compare variables before and after the intervention within each group. 

## 3. Results

Forty-four patients were recruited in this study and during the intervention. Five patients were excluded from the study due to non-cooperation or travel and withdrawal of two patients (case group) and three patients (control group). Therefore, thirty-nine patients with type 2 diabetes, 15 men and 24 women completed the study. The mean ± SD age and body mass index of the participants were 57.6 ± 8.7 years and 27.7 ± 4.5 kg/m^2^, respectively. 

According to Table 1, baseline demographic and anthropometric characteristics did not differ significantly between the two groups of the study population. Mean ± SD age of the patients was 58.9 ± 7.9 years in the intervention groups and 56.2 ± 9.4 years in the groups consuming placebo. Eight patients in the intervention group and seven in the placebo group were male and others were female (*p* = 0.83). In addition, mean ± SD body mass index was 26.4 ± 3.0 kg/m^2^ for the intervention group and 29.0 ± 5.5 kg/m^2^ for the placebo group.

A comparison of glycemic indices and inflammatory factors before and after of cinnamon consumption are presented in Table 2. Baseline levels of FPG, fasting levels of insulin, HbA1c, HOMA-IR, carboxymethyl lysine, total antioxidant capacity, and MDA in the patients of case and control groups were not significantly different. In Table 2, the intergroup differences of glycemic parameters and inflammatory indicators were also investigated in patients. In both case and control groups, after eight weeks of intervention, changes were observed in levels of FPG (−11.65 ± 29.34 vs. 8.57 ± 35.10 mg/dL), fasting insulin [2.05 (−1.62–5.45) vs. 1.20 (−2.40–4.70) mU/L], HbA1c (0.075 ± 1.51 vs. −0.15 ± 1.93), HOMA-IR [−0.03 (−1.50–1.97) vs. 0.68 (−0.73–1.50)], carboxymethyl lysine [0.00 (−2.00–6.00) vs. 2.00 (−5.00–8.00)], total antioxidant capacity (0.002 ± 0.11 vs. 0.006 ± 0.10 mmol), and MDA [0.00 (−3.12–0.52) vs. 1.05 (−1.57–2.96)] were not significantly different.

In addition, after eight weeks of intervention, there were no significant intra-group differences based on FPG, fasting levels of insulin, HbA1c, carboxymethyl lysine, total antioxidant capacity, and MDA in patients of either group (*p* > 0.05).

## 4. Discussion

The present study was designed to assess the effects of cinnamon intake on glycemic markers, AGEs, and inflammatory indicators in patients with type 2 diabetes. Results indicate that taking three gram of cinnamon supplements in patients had no beneficial effects on FPG, fasting insulin level, HbA1c, HOMA-IR, carboxymethyl lysine, total antioxidant capacity, and MDA after eight weeks of intervention.

The limited studies that have investigated the beneficial effects of cinnamon supplement on glycemic indicators, including FPG, insulin resistance, HbA1c, plasma insulin levels, and insulin sensitivity, in diabetic patients have documented controversial results. We found no association between using three grams per day of cinnamon supplement and serum levels of fasting insulin, FPG, HbA1c, and HOMA-IR, and results are consistent with the findings of the Steve et al. study, which indicated no significant change in FPG, HbA1c, or insulin levels after 1 g/day cinnamon supplementation intake for three months [15]. In addition, Vanschoonbeek et al. showed no significant improvement in FPG, oral glucose tolerance, or insulin sensitivity with 1.5 g/day cinnamon supplementation in diabetic patients [17]. It is probable that the intervention period of these studies was relatively short and non-significant changes in glycemic markers could become statistically significant with longer follow-up. Moreover, although another study reported a significant difference based on FPG in both the case and control groups, they found no significant intra-group or inter-group differences regarding HbA1c level [16]. As regards, the population of the Mang et al. study had poor controlled FPG at baseline, i.e., (mean = 200–300 mg/dL) compared to patients of our study (mean ≤ 190 mg/dL), indicating that diabetic patients with poor glycemic control may benefit more from positive effects of cinnamon supplementation. Therefore, contrary to Mang et al., we could not show a positive effect between cinnamon intake and level of FPG. 

In the Crawford et al. clinical trial study, contrary to our study, three months administration of one gram per day of cinnamon capsules to diabetic individuals showed improvement for HbA1c [14]. Considering that the duration of intervention can affect the amount of changes in HbA1c levels, differences in duration of treatment in our study (60 days) with Crawford et al. study (90 days) could likely be responsible for the discrepancy in findings. In addition, another study reported that consumption of three grams of cinnamon supplements for eight weeks in diabetic patients decreased levels of FPG and HbA1c in the intervention group, compared to the initiation of the study; however, as in our study, in the mentioned study, there were no significant differences in glycemic status indicators between the case and control groups at the end of intervention [18]. Overall, this heterogeneity of results of studies on the effect of cinnamon intake on glycemic markers can be explained by differences in multiple influential factors such as use of concurrent medications, duration of intervention, dosage form, cinnamon dose, ethnicity and BMI of the study population. Generally, considering the conflicting results on the probable beneficial effects of cinnamon supplement on glycemic indicators [13], more research proposed dose and duration of cinnamon supplementation in diabetic patients is obviously warranted and, at present, cinnamon cannot generally be recommended for treatment or control of type 2 diabetes.

In the current study, we also reported that 3 g/day cinnamon intake had no beneficial effects on MDA, antioxidant capacity, and AGEs. The findings of limited data in this context are poor and conflicting. In the only human study that has been done in this regard, intakes of cinnamon for six weeks showed no significant change in MDA levels of women athletes [24]. However, findings of our study are not in agreement with an experimental trial study of Amin et al., which showed that administration of cinnamon can modulate the oxidative stress, decreasing levels of MDA in rats [21]. In addition, in another animal study, oral administration of 200 mg/kg/day of cinnamon for seven days indicated protective effects on oxidative stress, by lowering the MDA levels and elevating antioxidant enzyme activities [22]. Differences in study type of these trials (in vivo animal studies) with our study (in vivo human study) and the administered dosage of cinnamon can account for the discrepant findings of these studies. 

To date, there is no in vivo trial that assessed the effect of cinnamon supplement on AGEs. Only one study examined the effect of cinnamon bark proanthocyanidins to prevent the formation of AGEs in a bovine serum albumin–glucose model, and results indicated the protective effect of cinnamon bark proanthocyanidins as a scavenge reactive carbonyl species in the inhibition of formation of AGEs [23]. 

Although some previous studies have provided us with interesting and promising data on the effects of cinnamon intake on inflammatory indicators or antioxidant factors, existing evidence about the beneficial effect of cinnamon supplementation on improving oxidative stress status and antioxidant capacity are very limited.

The strengths of our present study include its double-blind design and high participation rate and the least dropout. In addition, to our knowledge, this is the first clinical trial study in the Middle East and North Africa (MENA) region that assessed the effects of cinnamon intake on glycemic indicators along with inflammatory factors, and AGEs in diabetic patients. Limitations of the current study include the short follow-up period (eight weeks) and dose of the cinnamon supplementation (three grams per day). In addition, the intake of high doses of cinnamon supplementation [26] or a longer follow-up period of the previous trial [16] indicated significant effects on glycemic and inflammatory factors. Financial limitation and low compliance of patients (for longer intervention) were the most important reasons why we could not have a longer intervention period or another group with higher doses of cinnamon for comparing doses.

## 5. Conclusions

In conclusion, findings of this study revealed that an 8-week intervention of three grams of cinnamon supplement per day had no beneficial effects on FPG, insulin levels, HbA1c, HOMA-IR, carboxymethyl lysine, total antioxidant capacity, and MDA levels. Further studies are needed to better evaluate the impact of cinnamon intake on glycemic markers and indicators of oxidative stress or inflammation in diabetic patients.

## Figures and Tables

**Table 1:** Baseline lifestyle and anthropometric characteristics of the study population.

Characteristics	Total (*n* = 39)	Case Group (*n* = 20)	Control Group (*n* = 19)	*p*-Value
Age (years)	57.61 ± 8.70	58.90 ± 7.93	56.26 ± 9.46	0.43
Male (%)	38.5	40.0	36.8	0.83
Weight (kg)	75.41 ± 13.28	73.75 ± 10.74	77.15 ± 15.63	0.43
Height (cm)	164.89 ± 8.67	167.15 ± 7.38	162.52 ± 9.47	0.09
Body mass index (kg/m^2^)	27.70 ± 4.52	26.41 ± 3.06	29.02 ± 5.53	0.18

Data are presented as mean (standard deviation) for continuous variables and percent for categorically distributed variables.

**Table 2:** Comparison of variables in the groups of patients before and after the intervention.

Characteristics		Case Group (*n* = 20)	Control Group (*n* = 19)	*p*-Value *
Fasting blood glucose (mg/dL)	Before	183.85 ± 36.16	190.57 ± 70.58	0.71
	After	172.20 ± 44.86	199.15 ± 49.86	0.53
	Differences	−11.65 ± 29.34	8.57 ± 35.10	0.06
	*p* **	0.09	0.30	-
Fasting insulin (mU/L)	Before	9.85 (7.92–19.22)	10.60 (8.80–17.30)	0.86
	After	12.10 (10.65–18.45)	12.20 (9.30–14.20)	0.73
	Differences	2.05 (−1.62–5.45)	1.20 (−2.40–4.70)	0.86
	*p*	0.24	0.46	-
Homeostasis model assessment for insulin resistance (HOMA-IR)	Before	5.35 (2.97–9.22)	5.39 (2.64–6.98)	0.86
	After	6.00 (3.34–9.00)	6.16 (3.48–8.49)	0.83
	Differences	−0.03 (−1.50–1.97)	0.68 (−0.73–1.50)	0.42
	*p*	1.00	0.39	-
HemoglobinA1c	Before	10.04 ± 1.30	10.31 ± 1.86	0.59
	After	10.11 ± 1.49	10.30 ± 1.70	0.86
	Differences	0.075 ± 1.51	−0.15 ± 1.93	0.87
	*p*	0.83	0.97	-
Carboxymethyl lysine	Before	185.00 (178.50–188.75)	183.00 (178.00–189.00)	0.65
	After	187.00 (181.25–191.00)	185.00 (182.00–189.00)	0.39
	Differences	(−2.00–6.00)	2.00 (−5.00–8.00)	0.63
	*p*	0.44	0.44	-
Total antioxidant capacity (mmol)	Before	0.708 ± 0.12	0.710 ± 0.13	0.96
	After	0.706 ± 0.18	0.716 ± 0.13	0.80
	Differences	0.002 ± 0.11	0.006 ± 0.10	0.81
	*P*	0.93	0.78	-
Malondialdehyde	Before	5.73 (4.16–9.11)	5.21 (3.64–7.81)	0.47
	After	6.25 (4.29–7.29)	6.25 (4.69–7.81)	0.14
	Differences	0.00 (−3.12–0.52)	1.05 (−1.57–2.96)	0.08
	*p*	0.37	0.53	-

Data are presented as mean ± standard deviation (SD) or median (25–75 interquartile range) for continuous variables and percent for categorically distributed variables. * *p*-values are for the comparisons across groups, with the use of Student’s *t*-test for continuous variables, Mann–Whitney test for variables with non-normal distribution. ** *p*-values are for the comparisons between groups, with use of paired *t*-test for continuous variables, Wilcoxon test for variables with non-normal distribution.
